# Translational framework for implementation evaluation and research: implementation strategies derived from normalization process theory

**DOI:** 10.1186/s13012-025-01444-5

**Published:** 2025-07-27

**Authors:** Carl R. May, Alyson Hillis, Bianca Albers, Laura Desveaux, Anthony Gilbert, Melissa Girling, Roman Kislov, Anne MacFarlane, Frances S. Mair, Sebastian Potthoff, Tim Rapley, Tracy L. Finch

**Affiliations:** 1 Department of Health Services Research and Policy, London, School of Hygiene and Tropical Medicine, 15-17 Tavistock Place, London, WC1H 9SH UK; 2 Department of Health Services Research and Policy, London, School of Hygiene and Tropical Medicine, London, UK; 3 Institute for Implementation Science in Health Care, University of Zurich, Zurich, Switzerland; 4 Learning Health System Program Institute for Better Health, Trillium Health Partners, Toronto, Canada; 5 Royal National Orthopaedic Hospital, Stanmore, Middlesex UK; 6 Department of Nursing, Midwifery & Health, Northumbria University, Coach Lane Campus, Newcastle-Upon-Tyne, UK; 7 Manchester Metropolitan University, Business School, Manchester, UK; 8 WHO Collaborating Centre for Participatory Health Research With Refugees and Migrants, School of Medicine, University of Limerick, Limerick, Ireland; 9 General Practice & Primary Care, School of Health and Wellbeing, University of Glasgow, Glasgow, UK; 10 Department of Social Work, Education and Community Wellbeing, Northumbria University, Coach Lane Campus, Newcastle-Upon-Tyne, UK

## Abstract

**Background:**

Implementation strategies are deliberate systematic actions used to support the uptake of innovations in health and social care. While widely used taxonomies such as ERIC and EPOC have emerged from consensus exercises, few implementation strategies are explicitly derived from theory and tested against empirical data. This study develops a taxonomy of implementation strategies grounded in Normalization Process Theory (NPT), an implementation theory that explains how new practices become embedded and sustained.

**Methods:**

We conducted a qualitative evidence synthesis of studies that reported implementation projects informed by NPT. Studies were identified through citation tracking and database searches, screened using pre-specified criteria, and appraised for methodological quality. Using the NPT coding manual, we identified implementation mechanisms described in each study and translated these into candidate implementation strategies. These were then tested against all included studies through iterative qualitative content analysis.

**Result:**

Searches led to 9,147 references, and we then eliminated 5,708 duplicates. After title and abstract screening a further 1,443 were eliminated. Full text screening was undertaken with 1,996 papers, and 1,411 of these were eliminated. This left 585 papers subjected to quality assessment, of which 522 were eliminated. Finally, 63 papers were included in the review. Qualitative analysis of included papers yielded 24 general strategies linked to NPT’s theoretical constructs and 96 micro-strategies representing four domains of implementation activity: leadership, information, empowerment, and service user involvement. Each strategy was explicitly linked to an NPT construct.

**Conclusions:**

This study provides a theory-based and empirically grounded set of actionable implementation strategies. These are grounded in qualitative descriptions of implementation work. These strategies support practical decision-making across the planning, delivery, and sustainment phases of implementation, and offer context-sensitive guidance for adapting interventions to diverse settings. Unlike consensus-based taxonomies, these strategies are tied to observable mechanisms of action, enabling users to better understand and respond to the dynamic and socially organised nature of implementation. The NPT taxonomy of implementation strategies can support the design, tailoring, and operationalisation of implementation efforts across varied health and social care contexts.

**Supplementary Information:**

The online version contains supplementary material available at 10.1186/s13012-025-01444-5.

Contribution to the literature (i) This paper presents a taxonomy of implementation strategies explicitly derived from Normalization Process Theory (NPT).

(ii) The NPT Taxonomy of Implementation Strategies provides actionable implementation strategies derived from a systematic qualitative evidence synthesis of empirical studies informed by Normalization Process Theory.

(iii) The NPT Taxonomy of Implementation Strategies spans leadership, information, empowerment, and service user involvement, supporting strategy selection across varied contexts.

(iv) The NPT Taxonomy of Implementation Strategies links to theory-defined and empirically grounded mechanisms of action, offering a contrast to expert consensus taxonomies like ERIC and EPOC.

## Background

Implementation strategies are deliberate and systematic methods employed to support the implementation of innovations within health and social care settings. They constitute the ‘how to’ elements of implementation science [1]. Implementation strategies are expected to support the uptake of promising, evidence-based practices in order to achieve improved patient outcomes and more efficient service delivery [2, 3]. We define implementation strategies as: *activities that are embedded in the design and delivery of interventions with the expectation that they will improve implementation outcomes*. Building on this definition, we describe a set of implementation strategies derived from Normalization Process Theory (NPT).

Several taxonomies of implementation strategies have been developed to classify and support the use of these strategies. Among the most commonly used are the Cochrane Effective Practice and Organization of Care Group (EPOC) [4] taxonomy, the ERIC (Expert Recommendations for Implementing Change) taxonomies of implementation strategies [2, 5], and the Behaviour Change Wheel (BCW) [6, 7]. Founded on outstanding scholarship, these frameworks have each shaped how implementation is planned and reported but also face well-documented limitations. The EPOC taxonomy [4], developed within the Cochrane Collaboration, categorises strategies under professional, organisational, financial, and regulatory domains. It is designed to inform systematic reviews, and so is relatively inflexible and underrepresents the relational, informal, and emergent aspects of implementation processes. In addition, its categories are not linked to mechanisms of change, limiting its capacity to support theory-informed implementation planning or evaluation [8]. The ERIC framework [2, 5] consists of 73 discrete strategies identified through expert consensus meetings and a Delphi study. It is widely used in U.S.-based implementation projects and supports pragmatic, stakeholder-oriented planning. Nonetheless, critiques have pointed to its lack of theoretical coherence, definitional overlap between strategies, and limited explanatory power. Without links to mechanisms, ERIC risks being applied as a checklist rather than a theoretically coherent implementation approach [9]. The Behaviour Change Wheel [6, 7] addresses some of these limitations by linking individual behavioural determinants to intervention functions. It provides a strong theoretical foundation for behaviour-focused interventions. However, the BCW has been criticised for focusing primarily on individual-level behaviour change, with limited applicability to collective action or system-level implementation. Its complexity and resource requirements may also limit its utility in practice [10]. These limitations point to a gap in the implementation science literature: the lack of a strategy framework that is explicitly derived from theory, grounded in empirical data, sensitive to the collective social and organisational dimensions of implementation, and provides actionable strategies that can be implemented across different contexts.

Normalization Process Theory (NPT) [11–19] is an empirically grounded implementation theory [20] that ‘identifies, characterizes, and explains, mechanisms that motivate and shape implementation processes’ [21]. NPT starts from the position that an implementation process occurs ‘when one group of actors seeks to translate their strategic intentions into the everyday practices of others’ [21], and that ‘the essence of an implementation process is to be found in collective action and collaborative work’ [21]. The theory describes key mechanisms that appear to be universal, and that motivate and shape implementation processes. The aim of this study was to develop a set of actionable implementation strategies that are both theoretically derived from and empirically grounded in high quality qualitative research. NPT tells us important things about how implementation processes work and explains barriers and facilitators to successful implementation [11–19]. It therefore offers a useful foundation for implementation strategies. These can improve the likelihood of successful implementation by addressing both the technical and social dimensions of change. The aim, therefore, of NPT-based implementation strategies is to offer systematic guidance for the translation of strategic intentions into everyday practice [21]. The strategies presented in this paper are aimed at enabling the implementation [22] of evidence-based interventions and innovations in the organisation and delivery of health and social care *within* specific organizational settings. Because well-founded theories provide rational and replicable explanations of phenomena of interest, they support practitioners to better understand and think through the factors influencing implementation outcomes. Such explanations enhance the effectiveness and efficiency of implementation processes. The strategies we have developed here are intended to support implementation practitioners and researchers in the design and delivery of interventions across diverse health and social care settings. The paper addresses a gap in the literature around implementation strategies because it is explicitly derived from a coherent and validated implementation theory, and is also derived from empirical descriptions of implementation processes. This paper contributes a structured taxonomy of strategies that are tightly aligned with NPT constructs and that reflect the work of implementation as described in a systematic qualitative evidence synthesis of 63 peer-reviewed studies.

### Methods

**Fig. 1:**
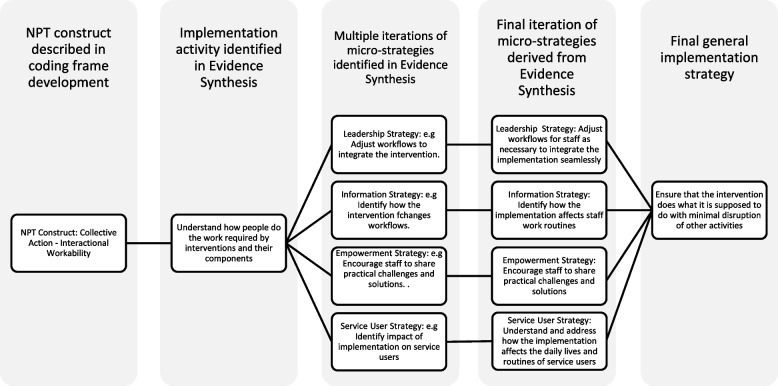
Sequence of research procedures to produce implementation strategies

This study links the development of a theory-informed coding manual for qualitative data [23], a qualitative evidence synthesis of empirical studies of implementation projects informed by NPT [24], and the development of a set of NPT-grounded implementation strategies. In Fig. 1 we show an example of the sequence of activities leading from an NPT Construct (Collective Action: Interactional Workability) defined in the coding manual, to micro and general strategies identified within the qualitative evidence synthesis.

### Qualitative evidence synthesis: of implementation studies informed by normalization process theory

#### Searches and citation analysis

Our searches updated those of our earlier review [18, 25]. Following the protocol [24], ‘we searched the Scopus and Web of Science bibliographic databases, and Google Scholar, to find publications that cited papers and chapters that developed or expounded the main constructs of NPT [26–32]; papers that developed NPT related methods or tools [33–35]; and citations of the NPT web-enabled on-line toolkit’ [36].

#### Screening

Title and abstract screening were performed online using Covidence™ software [37]. All potentially eligible citations were obtained in full text. Full text papers were independently screened by AH and CRM. Considerations of eligibility were resolved by discussion.

#### Inclusion and exclusion criteria

We included English language peer-reviewed health and healthcare-related journal articles published between 1 June 2006 (the date of publication of the first NPT paper) and 31 December 2021 that employed NPT either solely or in combination with some other theory to report results of (a) primary studies using qualitative or mixed methods, or (b), qualitative evidence syntheses. We excluded editorials or commentaries; protocols and other study designs; research monographs, theses or dissertations; books and book chapters; conference proceedings and abstracts; or webpages, blogs, or other social media. We also excluded peer-reviewed studies that solely report on quantitative study designs; that contained only nominal or passing references to NPT; that were restricted to methodological or theoretical discussions, or made theoretical or methodological recommendations; or that were reports of the application of NPT in settings other than those related to health, healthcare and social care.

#### Data extraction

Descriptive information was extracted, including authors, year of publication, health care problem addressed, study type and methods, data collection procedures, how NPT was used in the study and whether this had been pre-specified in the study protocol. An Excel file with the extraction instrument and complete information about all included studies is included in Supplementary Online Documentation. Procedures for the extraction of data for analysis are described below.

#### Quality appraisal

In additional work to identify papers that merited inclusion in the evidence synthesis, we identified those that either scored ‘high’ (16 or above) when their quality is assessed using the CASP [38] checklist, or that met the definitions developed by Kislov et al. [39, 40] of ‘theoretically informed’ papers that offer a rigorous non-descriptive analysis, and ‘theoretically informative’ papers that develop relationships between theoretical constructs or challenge theoretical propositions. All authors participated in quality appraisal.

### Qualitative data analysis: development and testing of NPT-informed implementation strategies

The NPT Coding Manual for qualitative research and instrument development [23] provided clear definitions of NPT constructs and subconstructs. It provided a coding framework for qualitative comparative analysis [41] that enabled us to identify potential implementation strategies, and present them in concise form as a theory-based matrix [42]. The matrix and coded data are presented in Online Supplementary Documentation (Appendix A). The matrix of strategies was then developed and elaborated through multiple iterations in discussion between CRM, AH, TR, and TLF, a process illustrated in Fig. 1. At the conclusion of the process of iteration we had a clear set of draft implementation micro-strategies and general strategies. Once an agreed set of descriptors of implementation strategies had been defined, we analysed peer reviewed studies of implementation projects informed by NPT [24] included in the synthesis. To do this we used the matrix of implementation strategies as a coding frame for qualitative content analysis [41] of the papers finally included in our qualitative evidence synthesis. All papers were then coded independently against the constructs in the NPT qualitative coding manual [23], in an initial round by CRM and AH. This was repeated independently in a second round by BA, TLF, AG, MG, FSM, SP, and TR. Results of this process are shown in Table 1.

**Table 1:** NPT construct, implementation micro-strategies and general implementation strategies

NPT Constructs	Implementation Micro-Strategies				General Implementation Strategies
	**Information Strategies (what do staff need to *know* to contribute to implementation?)**	**Empowerment Strategies (what needs to be *done* to equip staff to participate in implementation?)**	**Service User Strategies (how can service users *contribute* to implementation?)**	**Leadership Strategies (what do leaders need to do to *promote* implementation?)**	
***NPT Construct: Strategic Intention*** How do contexts shape the formulation and planning of interventions and their components?	Determine how information about context influences the goals of implementation [44, 45]	Involve a wide range of staff and stakeholders in the planning process to ensure that differences in perspectives and needs are taken into account [46–48]	Service users should contribute to tailoring the implementation to meet their specific needs and circumstances [49]	Develop a comprehensive plan for staff and service users that outlines the implementation’s objectives, taking into account the specific organizational context [45, 50–53]	Undertake collaborative work to build a coherent and inclusive implementation plan for the intervention
***NPT Construct: Adaptive Execution*** How do contexts affect the ways in which users can find and enact workarounds that make an intervention and its components a workable proposition in practice?	Identify aspects of the intervention that might require staff to improvise workarounds or adjustments during implementation	Encourage staff to develop and share workarounds that overcome contextual challenges [54, 55]	Elicit service users’ experiences and suggestions of practical workarounds that might not be apparent to healthcare providers [54, 56]	Establish an implementation framework for staff that allows for modifications and adaptations as the implementation is rolled out [47–49, 57–79]	Determine which components of the intervention can be adapted to better fit the target setting
***NPT Construct: Negotiated Capacity*** How do contexts affect the extent that an intervention and its components can fit, or be integrated, into existing ways of working by their users?	Engage with staff at all levels to understand their views on how the implementation can be integrated with current practices	Encourage staff to explore the compatibility of the implementation with their existing practices, structures, and capabilities	Elicit service user perspectives on the alignment of the implementation with their lifeworld and its routines	Collaboratively develop strategies with staff that align the implementation with existing workflows, modifying components where necessary to ensure a better fit [46, 47, 55, 57, 78, 80–83]	Engage stakeholders to ensure the intervention can be integrated in workflows in its target setting
***NPT Construct: Reframing organisational logics*** How do existing social structural and social cognitive resources shape the implementation environment?	Identify those features of the organization expected to affect implementation [59]	Involve key discussions about the organizational implications of implementation [49, 67, 75]	Assess service users’ expectations of care	Deliver targeted initiatives (like training programs or policy revisions) for staff to align implementation with organizational goals and structures [44, 64, 84, 85]	Identify features of the target setting that are likely to support implementation
***NPT Construct: Coherence building—Differentiation*** How do people distinguish interventions and their components from their current ways of working?	Show staff how the implementation differs from existing practices [44, 60, 86, 87]	Encourage staff to openly discuss perceived differences and their implications [73, 83, 84, 88–90]	Identify how implementation will lead to differences from service users’ current care routines [91]	Deliver targeted training sessions [84]	Clearly articulate how the new intervention improves upon current practices
***NPT Construct: Coherence building—Communal specification*** How do people collectively agree about the purpose of interventions and their components?	Clarify for staff the goals and expected outcomes of the implementation [46, 49, 62, 74, 77, 79, 87, 92, 93]	Facilitate group discussions amongst staff to develop shared understandings of implementation [55–57, 59, 61, 67–69, 80, 89, 94, 95]	Involve service users in consensus building about the goals of the implementation [58, 72]	Develop a consensus document outlining agreed objectives of implementation [45, 72, 89]	Establish and agree shared goals for the implementation process
***NPT Construct: Coherence building—Individual specification*** How do people individually understand what interventions and their components require of them?	Determine what each staff member needs to know to implement the implementation effectively [61, 73, 80]	Provide clear, role-specific guidelines and expectations for staff. [47, 62, 73, 85, 87]	Provide targeted information tailored to service user needs and circumstances [96]	Conduct individualized staff training sessions tailored to specific roles and responsibilities	Define and communicate individual roles and responsibilities related to the intervention
***NPT Construct: Coherence building – Internalisation*** How do people construct potential value of interventions and their components for their work?	Identify the value and benefits of implementation for staff [55, 73, 76, 87]	Share success stories about implementation amongst staff [53, 61]	Ensure that service users understand how implementation could improve their care or quality of life [97]	Create and disseminate materials for staff that illustrate the positive outcomes of implementation	Clearly identify the value of the intervention to staff
***NPT Construct: Cognitive participation – Initiation*** How do key individuals drive interventions and their components forward?	Identify key staff who will drive and champion for the implementation [85]	Prepare staff for leadership and train to motivate others [46, 47, 50, 51, 56, 81, 87, 98]	Identify service users who can act as champions for the implementation, sharing their stories and encouraging others to participate	Assign leadership roles to key staff and provide them with the necessary resources [49, 51–53, 56, 60, 82, 83, 88, 90, 99, 100]	Select and support key individuals who will drive the intervention forward
***NPT Construct: Cognitive participation – Enrolment*** How do people join in with interventions and their components? [48]	Understand the process by which staff can become involved in the implementation process	Make staff participation accessible and attractive [44, 48, 68, 69, 72, 85, 96]	Make it easy for service users to get involved, providing clear information and support as needed [45, 57, 60, 61, 74, 75, 79, 81, 83, 84, 86, 89, 91, 94, 96, 99]	Ensure commitment of staff [85, 87, 97]	Eliminate obstacles to participation in the implementation process
***NPT Construct: Cognitive participation – Legitimation*** How do people agree that interventions and their components are the right thing to do and should be part of their work?	Identify the ethical, professional, and organizational justification for the implementation [53, 70]	Encourage staff dialogue about the implementation’s legitimacy [49, 55, 62, 67, 79]	Show how participating in the implementation is legitimate from the service user perspective [59, 67, 72, 83]	Organize sessions for staff to discuss its alignment with professional standards and organizational goals [49, 94, 100]	Be clear about how, why, and for who the intervention is the right thing to do
***NPT Construct: Cognitive participation – Activation*** How do people continue to support interventions and their components?	Identify features of the implementation that lead to support from staff [70]	Ensure that staff leaders continuously engage with and support colleagues [75, 77]	Maintain ongoing communication with service users to keep them engaged with the implementation [94]	Implement regular audit and feedback mechanisms to track staff support [52, 56, 58, 87, 94, 101]	Develop strategies to maintain commitment among the implementation team
***NPT Construct: Collective action—Interactional workability*** How do people do the work required by interventions and their components?	Identify how the implementation affects staff work routines [52]	Encourage staff to share practical challenges and solutions.[53, 56, 63, 72, 77, 90, 93, 101]	Understand and address how the implementation affects the daily lives and routines of service users [54, 63, 72, 93]	Adjust workflows for staff as necessary to integrate the implementation seamlessly [48, 87, 93]	Ensure that the intervention does what it is supposed to do with minimal disruption of other activities
***NPT Construct: Collective action—Relational integration*** How does using interventions and their components affect the confidence that people have in each other?	Understand how the implementation affects relationships between staff [66, 100, 102]	Promote open communication amongst staff about relational dynamics [44, 83, 97]	Consider how the implementation impacts service users trust and confidence in healthcare providers [44, 67]	Facilitate problem solving activities and conflict resolution training for staff [92]	Foster positive and trusting interactions within the team
***NPT Construct: Collective action – Skill-set workability*** How is the work of interventions and their components appropriately allocated to people?	Determine the skill requirements for the implementation [56]	Assess and develop the required skills among staff [45–50, 52, 54, 57, 59, 61–69, 71–73, 75–78, 80–83, 85, 86, 90, 93, 95–97, 99–101, 103]	Provide service users with the knowledge and skills they need to participate effectively in the implementation [70, 71, 85]	Provide targeted training and redistribute tasks according to skill sets [45–50, 52, 54, 57, 59, 61–69, 71–73, 75–78, 80–83, 85, 86, 90, 93, 95–97, 99–101, 103]	Ensure staff have the skills required for effective implementation
***NPT Construct: Collective action – Contextual integration*** How is the work of interventions and their components supported by host organizations?	Gauge organizational readiness and resource availability [58]	Involve management in resource allocation and support [96]	Ensure that service user participation is actively supported by the host organisation [58, 92, 93]	Align organizational resources and policies to support the implementation.[45, 48, 55, 58, 59, 61, 77, 82, 92, 93, 95, 96]	Demonstrate organizational commitment and support for interventions
***NPT Construct: Reflexive monitoring – Systematisation*** How do people access information about the effects of interventions and their components?	Establish a system for monitoring the implementation’s effects [56, 60, 61, 64, 72, 84, 94, 99, 102]	Train staff to use monitoring tools effectively [65, 87]	Involve service users in monitoring the implementation and provide them with feedback on progress [71]	Implement and maintain a data collection and analysis system [56, 63, 67, 77, 79, 93, 95, 97, 102, 103]	Deploy systems to track progress and outcomes of the intervention
***NPT Construct: Reflexive Monitoring – Communal appraisal*** How do people collectively assess interventions and their components as worthwhile?	Determine criteria for assessing the implementation’s worth to staff [57, 68, 69, 81, 88, 89, 100, 102]	Involve staff in evaluating the implementation’s effectiveness [78, 90, 101]	Include service users in the evaluation of the implementation, ensuring their perspectives are considered in any assessments of its worth [58, 103]	Conduct regular review meetings and surveys for communal feedback [44, 46, 80, 99]	Create opportunities to continually improve the implementation process
***NPT Construct: Reflexive Monitoring – Individual appraisal:*** How do people individually assess interventions and their components as worthwhile?	Understand personal evaluations of the implementation	Encourage individual reflection and feedback [90]	Encourage and facilitate individual feedback from service users about their experiences with the implementation	Create channels for private feedback and personal reflection sessions	Create safe spaces for personal feedback about the implementation process
***NPT Construct: Reflexive Monitoring – Reconfiguration*** How do people modify their work in response to their appraisal of interventions and their components?	Identify necessary changes based on implementation appraisal by staff [49, 51, 56, 74–76, 78, 84, 96, 98, 100, 101, 103]	Allow staff to suggest and trial modifications [51–53, 55, 78, 86, 87, 95, 96, 102]	Be responsive to feedback from service users, and be prepared to make changes based on their experiences and suggestions [45, 63]	Facilitate an adaptable approach, revising practices based on staff feedback [45, 47, 50, 51, 60, 62, 66, 71–75, 77, 81–83, 90–92, 97, 99, 101]	Revise implementation process based on staff feedback
***NPT Construct: Intervention performance*** What practices have changed as the result of interventions and their components being operationalized, enacted, reproduced, over time and across settings?	Identify key metrics and indicators to measure changes in practices [47]	Encourage and enable staff to recognize and report changes in their practices	Identify service user reported outcomes of the implementation	Implement regular evaluation tasks for staff, to measure the implementation’s impact on practices [80]	Train staff to understand and contribute to the evaluation process
***NPT Construct: Relational restructuring*** How has working with interventions and their components changed the ways people are organized and relate to each other?	Determine how staff relationships and team dynamics have shifted due to the implementation	Facilitate open dialogue and feedback sessions for staff to express how their interactions and relationships have been affected [45]	Determine how the implementation has affected the relationships between patients, caregivers, and healthcare providers	Conduct workshops or team-building activities for staff to address and adapt to any changes in organizational relationships and structures	Update team structures to take account of change brought about by the implementation process
***NPT Construct: Normative restructuring*** How have working with interventions and their components changed the norms, rules and resources that govern action?	Assess how implementation has influenced the norms, rules, and resource distribution within the organization	Involve staff in reviewing and revising policies and norms to align with the implementation	Identify the impact on service users of changes in norms, or resources that affect them	Update organizational policies, procedures, and resource allocation strategies to reflect the changes brought by the implementation	Update organisational policies and guidelines to take account of changes brought about by the implementation process
***NPT Construct: Sustainment (normalisation)*** How have interventions and their components become incorporated in practice?	Identify the factors that contribute to the successful integration and routinization of implementation	Enable continuous engagement and ownership among staff to sustain the implementation	Work with service users to identify what factors will contribute to the successful incorporation of the implementation into their everyday lives	Integrate the implementation into standard operating procedures and ongoing training programs, ensuring it becomes a regular part of organizational practice	Be clear about how to decide if implementation has been successful, and for who

### Registration

The protocol for the evidence synthesis was published [24]. However, because the evidence synthesis focused on the development and application of an implementation theory it was not deemed eligible for inclusion in the PROSPERO Register of systematic reviews.

## Results

**Fig. 2:**
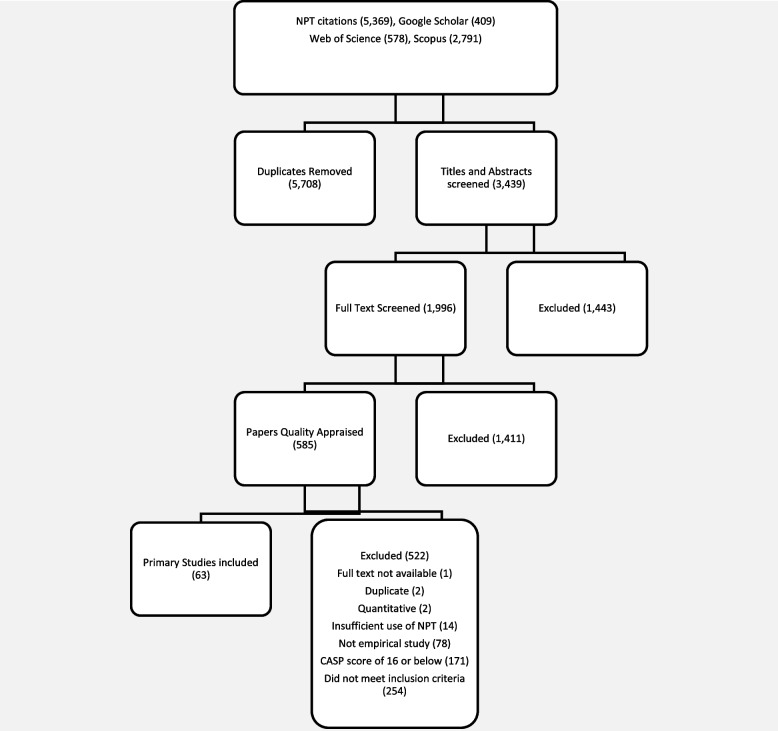
PRISMA flowchart

Searches for citations of papers and chapters that utilised the constructs of Normalization Process Theory [22, 26–32, 34, 43]; that developed NPT related methods or tools [33–35]; and citations of the NPT web-enabled on-line toolkit [36] led to 9,147 references, 5,708 of which could be eliminated as duplicates. As Fig. 2 shows, we then checked 3,439 titles and abstracts, and eliminated 1,443 of these, leaving 1,996 papers for full text screening; 1,411 of the latter were eliminated, leaving 585 full papers. Of these, 522 were excluded. Finally, 63 papers that either exceeded a CASP [38] score of 16 or were classified as theoretically informative [39, 40] were included in the review.

Analysis of papers included in the evidence synthesis showed that they identified implementation strategies that occurred *within* organisationally sanctioned implementation processes. In Table 1 we show key outcomes of our qualitative analyses. Each of the 24 constructs of Normalization Process Theory was matched to four implementation activities: leadership, information, empowerment, and service user involvement. From this analysis we derived 96 implementation micro-strategies, and 24 general implementation strategies. The sequence of analytic procedures by which these were produced is described in Fig. 1.

### Leadership strategies: what do leaders need to do to deliver implementation?

Leadership is fundamental to the successful organization and delivery of implementation projects [44]. Leaders’ roles in establishing an implementation framework for staff that allows for modifications and adaptations as the implementation is rolled out was centrally important [45–70]. Strategies that promoted consensus about the objectives of implementation [60, 71, 72] were linked to aligning implementation with existing workflows, modifying intervention components where necessary to ensure a better fit [45, 62, 69, 73–78], and to aligning organizational resources and policies to support implementation [46, 47, 49, 52, 67, 68, 71, 76, 78–85]. Not surprisingly, targeted training and redistribution of tasks was seen as a core implementation strategy [45, 47, 49–57, 59–63, 65–69, 71, 73–77, 80–82, 84–95]. Relevant strategies included assigning leadership roles and resources to others [48, 61, 76, 77, 86, 88, 89, 92, 96–99], taking into account the specific organizational context in which these roles are worked out [71, 87, 92, 98, 99]. Orienting leadership work in this way was seen as facilitating adaptation, revising practices based on staff feedback [48, 50, 54, 59, 60, 62–65, 68, 71, 75–77, 79, 86–88, 94, 95, 98, 100], ensuring its alignment with professional standards and organizational goals [61, 89, 101], along with adjusting workflows for staff as necessary to integrate the implementation seamlessly [67, 80, 102]. Strategies like audit and feedback mechanisms may influence the conduct of implementation processes [46, 92, 95, 97, 101, 102], but they rely on leaders committing to maintaining a system for data collection and analysis about the progress of implementation projects [51, 55, 68, 70, 80, 82, 90, 94, 97, 103]. These might include review meetings and surveys to obtain feedback [73, 74, 83, 86], enacting problem solving activities and conflict resolution [79], and seeking ways to secure the continued commitment of participating staff [85, 94, 102].

### Information strategies: what do staff need to know?

Determining what staff need to know about the planned process of implementation is a strategically important problem, in part because shared knowledge about action is a fundamental requirement for its coordination [104]. However, although there were many references to training and education to equip staff to operationalise interventions, there were surprisingly few references to attempts to determine what staff need to know to effectively perform implementation [49, 63, 74], and to understand how the implementation differs from existing practices [48, 83, 91, 102]. There was an emphasis on staff knowing about and understanding the goals and expected outcomes of an implementation process [50, 61, 64, 68, 70, 73, 79, 80, 102], along with its value [63, 66, 78, 102] and hence its justification [58, 99], along with the ways that it might affect relationships between staff [54, 58, 89, 103] and their routines and skill requirements [97]. More mechanistically, studies proposed that it was important to establish a system for monitoring the implemented intervention’s effects [48, 49, 52, 60, 84, 86, 97, 101, 103], for assessing its worth to staff [45, 56, 57, 72, 75, 89, 96, 103], and for making necessary changes based on implementation appraisal by staff [61, 64–66, 69, 81, 84, 89, 90, 95, 97, 98, 105].

### Empowerment strategies: how can staff participate in implementation?

Information on its own is an insufficient foundation for coordinated translational action. Strategies that build empowerment emphasise the development of required skills among staff [32, 45, 47, 49–57, 59–63, 65–69, 71, 73–77, 80–82, 85–95]. These strategies prepare participants for leadership and can include training to motivate others [62, 73, 75, 87, 97, 98, 102, 105]. An important feature of empowerment strategies was the facilitation of group discussions amongst staff to develop shared understandings of implementation processes [45, 47, 49, 55–57, 72, 74, 78, 82, 97, 101]; to openly discuss perceived differences and their implications [63, 72, 77, 84, 88, 96]; to develop and share workarounds that overcome contextual challenges [51, 60, 68, 78, 80, 88, 93, 95, 97, 99]; and to engage in discussions about the organizational implications of implementation [55, 61, 65]. Whilst training and adaptation play an important part in empowering participants in implementation processes, other strategies also stem from them. Here, ways need to be found to involve a wide range of staff and stakeholders in the planning to ensure that differences in perspectives and needs are taken into account [62, 67, 73], and roles and expectations are understood and accepted [50, 62, 63, 85, 102]. Equally, staff participation needs to be made accessible and attractive [56, 57, 60, 67, 81, 83, 85], perhaps through shared success stories [49, 99], and discussion about the legitimacy of an implementation process [50, 55, 61, 70, 78]. Participating staff can be further empowered by creating opportunities to suggest and explore modifications to implementation processes [69, 78, 81, 82, 91, 92, 98, 99, 102, 103].

### Service user strategies: how can intervention beneficiaries contribute to implementation?

Involvement of service users and caregivers in studies included in the review was variable and often cursory, reflecting the differences in research cultures and healthcare systems of included countries. Included studies emphasised the need to make it easy for service users to get involved, providing clear information and support as needed [45, 48, 49, 64, 65, 70–72, 75, 77, 81, 84, 86, 91, 100, 101]. This included involving service users in consensus building about the goals of the implementation [46, 60], and exploring how participation is legitimate from the service user perspective [47, 55, 60, 77]. Reciprocal participation from service users and caregivers included exploring their experiences and suggestions of practical workarounds that might not be apparent to healthcare providers [93, 97]. These took into account and addressed how the implementation affected the daily lives and routines of service users [51, 60, 80, 93]. Service users needed to be provided with targeted information and skills required to participate effectively in the implementation [58, 59, 85], and their participation had to be actively supported by the host organisation [46, 79, 80]. Also important was evidence of responsiveness to feedback from service users, and willingness to make changes based on their experiences and suggestions [51, 71].

## Discussion

Normalization Process Theory offers an approach to understanding how implementation work is organized, enacted, and sustained. By identifying empirically observed implementation mechanisms, NPT provides a foundation for developing actionable strategies. Our strategy development process maintained a clear commitment to the core constructs of the theory, ensuring that each proposed strategy could be directly traced to an observed mechanism within empirical studies. The second contribution of our development process is the structuring of strategies around key operational foci: leadership, information, empowerment, and service user engagement. These categories emerged inductively through analysis of empirical material, reflecting how implementation work is actually organized rather than how it is assumed to occur. Notably, service user focused implementation strategies are relatively underdeveloped in the literature, suggesting an important area for further research and refinement. Unlike EPOC [4], ERIC [2, 5], and the Behaviour Change Wheel (BCW) [6, 7], which build on expert consensus meetings and Delphi studies, our approach to the development of implementation strategies builds directly on theory-informed empirical observations of reported implementation processes. In doing so, it offers an alternative foundation for the design and delivery of implementation strategies that aims for both greater theoretical coherence and empirical grounding.

Existing strategy taxonomies are founded on outstanding scholarship and have been highly influential in shaping implementation practice. EPOC’s [4] focus on health systems interventions, ERIC’s categorization of strategies to address known barriers [2, 5], and the BCW’s emphasis on linking behavioural determinants to intervention functions [6, 7], have each advanced the field. This introduces a potential weakness, around construct validity and the risk of strategies being artefacts of expert classification and interpretation rather than reflections of real-world dynamics. Our approach directly addresses this problem by linking strategy development to mechanisms that are consistently revealed across empirical studies of implementation processes.

### Using NPT-derived implementation strategies

During the delivery phase of an implementation project, the strategies described in this paper could be selected, tailored, and operationalized through a detailed action plan that is founded on a structured method—for example, implementation mapping [106, 107]—that specifies not just methods to select strategies to be executed, but also those responsible for executing them, the resources required to support these activities, and the timelines for each strategy [2]. Throughout an implementation project, process evaluations can explore the effectiveness of implementation strategies in real-time, allowing for continuous adjustment and refinement. This dynamic approach ensures that strategies remain relevant and effective, enhancing the sustainability of the implementation. By integrating these strategies into the design and delivery phases, implementation facilitators and others can foster a comprehensive and adaptable implementation process, thereby increasing the likelihood of successful and sustained change.

### Strengths and limitations

A key strength of this study is that it starts from structured analysis of implementation mechanisms rather than experiential consensus meetings on interventions. The systematic use of the NPT coding manual [23] as a foundation for qualitative comparative analysis to extract and structure the strategies ensures transparency and, as far as is possible in qualitative investigation, replicability. The analysis addresses a recognised gap in the literature by providing a theory-based taxonomy of implementation strategies, linked to mechanisms of action. This adds value to their practical relevance and ensures that they are informed by descriptions of implementation work drawn from diverse healthcare contexts. The presentation of both micro-strategies and general implementation strategies supports flexible application across varied implementation settings and resource conditions. However, there are several limitations to our study. The qualitative evidence synthesis was of studies not designed to describe implementation strategies. This means that strategy identification depended on interpretive coding rather than direct observation or reporting. Furthermore, the evidence synthesis focused exclusively on studies that employed NPT. A risk in qualitative studies like this is hidden interpretive bias resulting from philosophical commitment to a particular theory, model or framework in implementation science. To counter this, three authors (BA, RK, SP) were recruited to this study because their disciplinary and theoretical allegiances lie elsewhere. It is possible that if we had used a different conceptual framework, or reviewed different reports of empirical studies, we might have produced a different set of implementation strategies.

## Conclusion

By deriving implementation strategies from Normalization Process Theory and grounding them in empirical observations drawn from papers included in a qualitative evidence synthesis, this paper provides a structured taxonomy of implementation strategies. Ensuring that theory-derived strategies can be linked to empirically observed practices ensures that mechanisms of change can be identified and understood. Implementation strategies should support both *thinking* and *doing* by leaders, practitioners, and researchers, as they design interventions and implementation projects. The contexts of implementation research and practice are complex, dynamic, and emergent, and structured through professional, organisational, and political hierarchies of power, influence, and control. The implementation strategies identified in this paper may not only help managers, practitioners, researchers, patients, and caregivers as they think through delivering change, but they might also represent day-to-day tools for responding to complexity and emergence as change takes place. Therefore, focusing on activities that are critical for supporting implementation enables us to offer a more nuanced understanding of the ways that strategies are operationalised according to the specific needs and contexts of implementation practitioners.

## Supplementary Information

Supplementary Material 1

## Data Availability

All data generated or analysed during this study are included in this article or in the attached online supplementary documentation.

## Abbreviations

BCW: Behaviour Change Wheel
CASP: Critical Appraisal Skills Programme
EHR: Electronic Health Record
ERAS: Enhanced Recovery After Surgery
ERIC: Expert Recommendations for Implementing Change
EPOC: Effective Practice and Organization of Care
ePRO: Electronic patient reported outcome measure in renal service
G-AP: Goal setting and action planning
GPs: General Practitioners
NIHR: National Institute of Health and Social Care Excellence
NPT: Normalization Process Theory
PCT: Primary Care Trust
PRISMA: Preferred Reporting Items for Systematic reviews and Meta-Analyses
PPI: Patient and Public Involvement
SSC: Surgical Safety Checklist
VIG: Video Interaction Guidance
